# The importance of nutrient hotspots for grazing ungulates in a Miombo ecosystem, Tanzania

**DOI:** 10.1371/journal.pone.0230192

**Published:** 2020-03-30

**Authors:** Gabriel Mayengo, Alex K. Piel, Anna C. Treydte

**Affiliations:** 1 Department of Sustainable Agriculture, Biodiversity Conservation and Ecosystems Management, The Nelson Mandela African Institution of Science and Technology, Arusha, Tanzania; 2 Department of Wildlife Management, College of African Wildlife Management, Mweka, Moshi, Tanzania; 3 School of Biological and Environmental Sciences, Liverpool John Moores University, Liverpool, United Kingdom; 4 Agroecology in the Tropics and Subtropics, Hans Ruthenberg Institute, University of Hohenheim, Stuttgart, Germany; Texas State University, UNITED STATES

## Abstract

While movement patterns of grazing ungulates are strongly dependent on forage quality their use of nutrient hotspots such as termite mounds or grazing lawns has rarely been quantified, especially in savanna ecosystems where soil-nutrient quality is low. Additionally, few experiments have been conducted to determine the role of termite mound- and grazing lawn-derived soils in improving forage quality in the field. We studied wild ungulate grazing activities around ten termite mounds, six grazing lawns and their respective control sites in a Miombo system of Issa Valley, western Tanzania, in the same system. We used indirect observations (i.e., dung, tracks) to identify seasonal and spatial variations in habitat use of various wild mammalian grazers. Grazer visitation rates were nine and three times higher on termite mounds and grazing lawns, respectively, compared to control sites. During the rainy season, termite mounds were more frequently used than grazing lawns while the latter were used more often during the dry season. In an additional pot experiment with soils derived from different areas, we found that *Cynodon dactylon* in termite mound-derived soils had twice as high Nitrogen and Phosphorous contents and biomass compared to grasses planted in grazing lawn soils and control site soils. We highlight that both termite mounds and grazing lawns play a significant role in influencing seasonal nutrient dynamics, forage nutrient quality, habitat selectivity, and, hence, grazing activities and movement patterns of wild ungulate grazers in savannas. We conclude that termite mounds and grazing lawns are important for habitat heterogeneity in otherwise nutrient–poor savanna systems.

## 1. Introduction

Nutrient hotspots [*sensu* 1,2] are crucial elements in the feeding ecology of wild herbivore species in heterogeneous savanna systems [3]. Grazers like impala (*Aepyceros melampus*) and Thomson’s gazelle (*Eudorcas thomsonii*) frequently graze on nutrient hotspots [4]. Nutrient thresholds for metabolism maintenance in herbivores are often only reached through specific forage preferences [5,6]. Termites *(Macrotermes* spp*)* act as soil ecosystem engineers by enhancing decomposition and mineralization processes, hence promoting soil turnover and influencing soil nutrient distribution [7]. However, less is known about how strongly their mounds contribute to vegetation quality, to associated ungulate foraging activity, and, consequently, to the overall heterogeneity of the landscape in the field [8]. Rainfall sweeps remarkable amounts of this nutrient-rich soil to immediate surroundings [9], which are then often colonized by grass species of high nutrient demand [10]. High forage quality and high plant diversity due to increased soil nutrients around termite mounds, therefore, is expected to lead to higher grazing pressure by ungulate mammals [11,12]. A locally high grazing pressure has also been associated with grazing lawns, which are characterized by the presence of short grass species, as well as maintained and influenced by high grazing intensity [4,13–15]. Within grazing lawns, grazers keep grasses short and the freshly regrown grasses with low stem proportions are highly palatable [4,16,17]. These lawns are normally self-maintaining as grass quality increases through the cycle of grazing-dung deposition [11,15].

Various mammalian grazers such as the wildebeest (*Connochaetes taurinus*) are attracted by short green grass of high nutrient content [18–20]. While the importance of grazing lawns for herbivores has been demonstrated [15,21,22] few experiments exist that quantified the extent to which these lawns attract grazers in nutrient poor areas [8]. In addition, the extent to which the grass layer around these hotspots (i.e., termite mounds and grazing lawns) is consumed by grazers is poorly understood. Particularly in the Issa Valley, where termite mounds are important for chimpanzee *(Pan troglodytes schweinfurthii)* foraging activities [23,24], little is known about the importance of termite mounds for sympatric mammalian herbivores. Moreover, termite mound and grazing lawn soils have never been compared experimentally simultaneously for their potential in enhancing grass growth and nutrient quality in the field. We used indirect observations to quantify wildlife presence and field vegetation assessment and experiments to investigate nutrient hotspots in a Miombo system at the Issa Valley, Tanzania. We analysed these hotspot grazing systems against control areas to test the following hypotheses: (a) Grass and soil nutrients in hotspot areas are richer than in their surroundings. Further, we expected that (b) grazing ungulates will forage more frequently in hotspot areas than in the surroundings and that (c) beneficial grass properties (height, cover and greenness) and high nutrient contents are the main attractants for foraging herbivores. In addition, we predicted that (d) termite mound and grazing lawn soils enhance grass biomass in otherwise nutrient-poor savanna landscapes, and that (e), nutrient hotspots will be used differently over the seasons by grazing mammals.

## 2. Material and methods

### Study area

The Issa Valley (05° 23 S 30° 35 E), which covers about 90 km^2^, is found in western Tanzania and consists of wide valleys and steep mountains from 900–1800 masl [25]. The dominant vegetation in the area is Miombo woodland (*Brachystegia* and *Julbernardia* species), with interspersed swamps, grasslands, thickets and closed canopy forests [24,25]. Issa is characterized by the wet season (>100 mm of rainfall monthly) from November-April and the dry season from May-October [<100 mm rainfall;24]. Some common mammalian ungulate species found in the study area are Lichtenstein’s hartebeest (*Alcelaphus lichtensteinii*), roan antelope (*Hippotragus equinus*), reedbuck (*Redunca redunca*), klipspringer (*Oreotragus oreotragus*), blue duiker (*Philantomba monticola*), common duiker (*Sylvicapra grimmia*), buffalo (*Syncerus caffer*), bushbuck (*Tragelaphus scriptus*), warthog (*Phacochoerus africanus*) and bushpig (*Potamochoerus larvatus*) [26]. The area does not have formal protective status [26] and is surrounded by recent villages established in the 1970s [25] where small scale logging, agriculture, wildlife snaring and camping activities have been documented [24,26]. During our survey, we also encountered local people harvesting *Orchid* species (pers.obs). A permanent research presence in Issa Valley was established in 2008 by the Greater Mahale Ecosystem Research and Conservation (GMERC) Project [25]. This study complied with Tanzanian Wildlife Research Institute (TAWIRI) ethical regulations permission was granted from TAWIRI and the Tanzanian Commission for Science and Technology (COSTECH).

### Data collection

We selected ten active termite mounds, covered with grass, that were not close to water bodies (i.e., more than 100 m away from water source) or from big trees that were on average (±SE) more than 9.0 ± 0.3 m tall and had a canopy radius of 5.5 ± 0.2 m [27] to avoid potential confounding factors. We further selected ten respective control sites 100 m away from the mounds [28], away from big trees. We established transects radiating away from each termite mound centre in all four cardinal directions (N, S, E,W), placed a 1 x 1 m^2^quadrat at 2 m, 12 m and 22 m distance away from the base of the mound [following 28]. In each quadrat, we surveyed grass communities, i.e., species and their respective basal cover, on and around the mound [29,30]. Additionally, in each quadrat, we measured standing biomass by harvesting all grasses to ground level and recording their dry weight between May 2016 and October 2017. Grass biomass was measured in February, May and September. In each quadrat, there were 4 sub-quadrats of 50 x 50 cm^2^. At each sampling event, a new location of the four subplots was used. We followed the same procedure for six grazing lawns at distances of 20 m, 40 m and 60 m away from the grazing lawn centre and their respective control sites were at a distance of 100 m away from the grazing lawn edge. Grass identification in all study plots was done with the assistance of a botanist and published literature [31]. We measured vertical grass height at four different points within each sampling quadrat, thereafter averaged the height measurement for all sampling plots [19]. We assessed grass tuft use and estimated use as a percentage in all quadrats [1,32]. Grass tuft usage was estimated by placing a quadrat of 1 m^2^, within which we counted all tufts and visually estimated (in %) the number of grass tufts that were eaten partially or fully by the mammalian herbivore [27]. Grass preference indices were assessed within each quadrat as 0 (no grass available), 1 (no grazing, i.e., none of the grass tufts showed bite marks), 2 (moderate grazing/very light grazing, i.e., partially eaten), 3 (heavy grazing) and 4 (intense grazing) [13]. Additionally, we visually assessed grass greenness as dry grass (a), pale green (b), green (c) and deep green (d) [20,33,34], (see supplementary information). However, ten termite mound areas and control sites in Miombo vegetation as well as some areas in grazing lawns edges were heavily affected by fire from July 2017 onwards and grass assessment was not possible in these sites. Grass assessments were done in February, May and September. However, for termite mounds, September data were not included in the analysis due to the effect of fire. In grazing lawns, which were moister than termite mounds in September, only edges were affected by fire and, hence, February, May and September data were included in the analysis (see also Table 1).

**Table 1:** Sample sizes (N) for laboratory analyzed samples from manipulation experiment, observation experiment and *C*.*dactylon* experiment, indicating number of samples that were involved in our grass analysis, their control and their initial measurement before fertilization. In the manipulation experiment column, we report the number of grass samples that were analyzed in each treatment, while in the field plots column, we report the number of soil and grass samples around each termite mound, grazing lawn as well as their respective controls that were involved in the analysis. Furthermore, in our *C*.*dactylon* pot experiments column, we report the number of grass samples that were analyzed from pots with soils derived from grazing lawns, termite mounds and controls. Fertilizer was applied once in the month of January, 2017. Laboratory analyses of grass and soil samples included nitrogen and phosphorus contents. GL = grazing lawn, TM = termite mound.

Manipulation experiment lab samples		Field plots lab samples				*Cynodon dactylon* experiment lab samples	
Treatment	Grass	Termite Mound		Control		Treatment	Grass
NPK	10	Grass	Soil	Grass	Soil	GL derived soil	10
Control	5	15	8	15	8	TM derived soil	10
Before NPK	5					Control soil	10
		**Grazing Lawn**		**Control**			
		Grass	Soil	Grass	Soil		
		15	4	15	4		

Additionally, to tease apart the factors that might contribute to the attractiveness of nutrient hotspots, we created experimental plots in the field that were at least 100 m away from shade or water bodies to avoid confounding factors. Ten plots of 5 x 5 m^2^ each were subjected to three treatments: i) fertilized with NPK fertilizer (ETG Input NPK 17-17-17 400 g/m^2^) irrigated with 10 l/m^2^ of water once; ii) cut to ground level and iii) a control treatment (no treatment), summing up to 40 experimental plots in total. Fertilizer was applied and irrigated once (as in treatment iii) to avoid pellets being eaten by animals. In addition, we sampled the most common grass in the area, *Hyparrhenia hirta*, before and after the experiment to assess its nutrient status (Nitrogen (N) and Phosphorus (P) content). *Hyparrhenia hirta* is common to other similar areas [35] and frequently grazed by herbivores [36]. Further, we visually assessed the extent of grazing (as % of grass tufts eaten, see above) on each plot [37]. The presence of different wildlife species was determined by recording cumulative dung depositions (graded as 1-fresh, 2-recent, 3-old) and tracks, i.e., footprints [38,39], whereby a series of tracks for an animal that was moving in one direction was considered as one event. After recording evidence, we removed signs to avoid re-counting. We identified dung and tracks using [40], with the assistance of experienced Tanzanian field assistants in all plots. From five termite mounds, five grazing lawns and five control sites, we collected composite soil samples, mixed the soil of each category separately, distributed each composite sample across 10 different pots and planted a common grass species, *Cynodon dactylon*, in the soils derived from those three categories, making a total of 30 pots (Table 1). *Cynodon dactylon* was chosen for pot experiments because of its seed availability, the fact that it is commonly found in savanna habitats [41] and due to its ability to grow in soils with a wide PH range [42].

### Grass and soil analysis

We collected grass and soil samples from ten termite mounds, six grazing lawns, their respective control sites, experimental plots in the field and samples from the pot experiment for measurement of available N and P contents, i.e., essential nutrients for ungulates [1,34].

Samples were air dried [43,44] and transported to the Core Facility Centre, Hohenheim University, Germany. Soil samples were ground using mortar and pestle, while grass samples were ground using Fritsch’smill with a sieving ring of 0.5 mm, digested under a microwave ultra clave [45] and introduced into ICP-OES (Vista Pro) [46–48] to determine total N and P contents. We analyzed a total of 101 grass and 24 soil composite samples from 332 samples in total from 122 plots (Table 1). The laboratory protocol that was followed can be found at dx.doi.org/10.17504/protocols.io.bb7tirnn.

### Data analysis

Grass species richness and diversity indices averaged for termite mounds, grazing lawns and their respective controls were compared using t-test in Paleontological Statistics (PAST) software [49]. Grass height and biomass from areas on termite mounds and grazing lawns were compared against 2 m, 12 m and 100 m, and for grazing lawns at 20 m, 60 m and 100 m, respectively, using one-way ANOVA. Grass greenness and basal cover on termite mounds and grazing lawns was compared with that of controls using t tests and one-way ANOVA. Further, a Generalized Linear Mixed Model (GLMM) was also applied to evaluate the effect of site (random factor), location (hotspot vs control) and season (wet vs dry) and their interactions with grass biomass. Grass preference index scores of different species were averaged for each grass species separately, divided by the highest grazing score (= 5), thereafter computed into percentage. A GLMM was further used to evaluate the effect of nutrient hotspots site i.e. hotspot vs control and their distance i.e. closeness to the nutrient hotspot and their interaction with nutrient content (N). We tested the interaction between location, distance, season and nutrient availability against tufts usage estimates using GLMM. *C*.*dactylon* grass height and nutrient contents after 61 days was compared across pots with termite mound, grazing lawn and control soils using one-way ANOVA. In the fertilizing experiment, grass N and P content levels were measured before setting the experiment, and after application of fertilizer, and then compared across treatments using a one-way ANOVA. We compared herbivore presence (tracks and dung) across fertilized, clipped [50] and control plots using GLMM. Grass height and tuft usage estimates were compared across fertilized, clipped and their control plots, as well as termite mounds, grazing lawns and their respective controls using a one-way ANOVA. Herbivore presence (tracks and dung) was compared between hotspots and controls as well as over dry and wet season using one-way ANOVAs. Tukey’s Post-hoc tests were used in all statistical tests, with significant levels set at α = 0.05. The software used was Origin Pro 8 [51] and SPSS version 20 [52].

## 3. Results

### 3.1 Grass and soil nutrient characteristics on and around hotspot areas are richer than in their surroundings

In total, 17 grass species were found across the study plots, of which *Hyparrhenia hirta*, *Andropogon gayanus*, *Digitaria* spp, *Themeda triandra*, *Panicum repens* and *Oryza longistaminata* were most frequently encountered. Grass species richness differed slightly between termite mounds and controls but not between grazing lawn and control plots (Tables 1 and 2), while grass species diversity indices did not differ (Tables 3 and 4).

**Table 2:** Sample sizes (N) for plots that were involved in the manipulation experiment, observation field plots and *C*.*dactylon* experiment. Manipulation experiment includes plots that were fertilized and their controls, clipped plots and their controls. The observational study shows plot numbers around termite mounds, grazing lawns and their controls. Further, *C*.*dactylon* pot experiments show the number of pots from grazing lawn derived soils, termite mound derived soils and control soils. Experiment duration has also been indicated at the bottom.

Manipulation experiment		Observation Termite Mound (TM)		*Cynodon dactylon* experiment	
Treatment	N	TM (N)	Control (N)	Treatment	N (pots)
NPK and irrigated	10	10	10	GL soil	10
Control for NPK	10			TM soil	10
Clipped	10	**Observation Grazing Lawn (GL)**		Control	10
Control for clipped	10	**GL (N)**	**Control (N)**		
		6	6		
Experiment duration:12 months		Observation timings: For TM: 12 months For GL:12 months		Experiment duration:61 days	

**Table 3:** Summary of variables assessed for grass and soil samples on termite mound sites vs control sites with their respective sample size (N), *F* value, *P* value, *t* value and degree of freedom (df) values. Grass height, biomass, cover, N and P values are shown as mean ± SD. Data were collected from May 2016 to October 2017 in the Issa Valley, Tanzania.

Variable	Termite mound		Control	N	*F*	*P*	df
Grass species richness		13±7	14±3	45	3.34	0.027	(3,44)
Diversity index (Shannon)		2.14	2.08	40	t = 0.29	0.773	(37.2)
Height (cm)		55±5	39±3	40	55.42	<0.0001	(3,36)
Dry biomass (g/m^2^)		238±42	172±25	40	2.20	0.104	(3,36)
Grass greenness score		3.6±0.3	2.5±0.3	10	t = 12.07	<0.0001	(9)
Basal cover (%)		24±11	18±8	26	0.67	0.644	(5, 25)
Grass N (%)		0.26±0.04	0.13±0.02	17	25.55	<0.0001	(3, 16)
Soil N (%)		0.21±0.04	0.09±0.04	13	13.57	0.0004	(3,12)
Grass P (mg/kg)		238±82	113±36	17	13.29	0.0001	(3,16)
Soil P (mg/kg)		236±62	107±13	13	7.96	0.0034	(3,12)

**Table 4:** Summary of variables assessed on grazing lawn sites vs control sites with their respective sample size (N), *F* value, t value, *P* value and degree of freedom (df) values. Data were collected from May 2016 to October 2017 in the Issa Valley, Tanzania. Grass height, biomass, cover, N and P values are shown as mean ± SD. N = sample size for the different analyses.

Variable	Grazing lawn	Control	N	F	P	df
Grass species richness	11±3	11±3	57	0.053	0.983	(3, 56)
Diversity Index (Shannon)	1.808	1.746	73	t = 0.32	0.751	(71.8)
Height (cm)	47±3	60±3	21	17.46	<0.0001	(3,20)
Dry biomass (g/m^2^)	214±38	278±43	21	0.77	0.524	(3,20)
Grass greeness score	2.5±0.58	1.8±0.4	6	t = 2.12	0.086	(5)
Basal cover (%)	20±31	20±21	25	0.26	0.932	(5,24)
Grass N (%)	0.30±0.040	0.15±0.03	17	18.42	<0.0001	(3,16)
Soil N (%)	0.81±0.01	0.23±0.03	8	13.67	0.014	(3,4)
Grass P (mg/kg)	424±178	222±59	20	4.68	0.015	(3,16)
Soil P (mg/kg)	543±106	211±18	5	8.14	0.035	(3,4)

**Fig 1:**
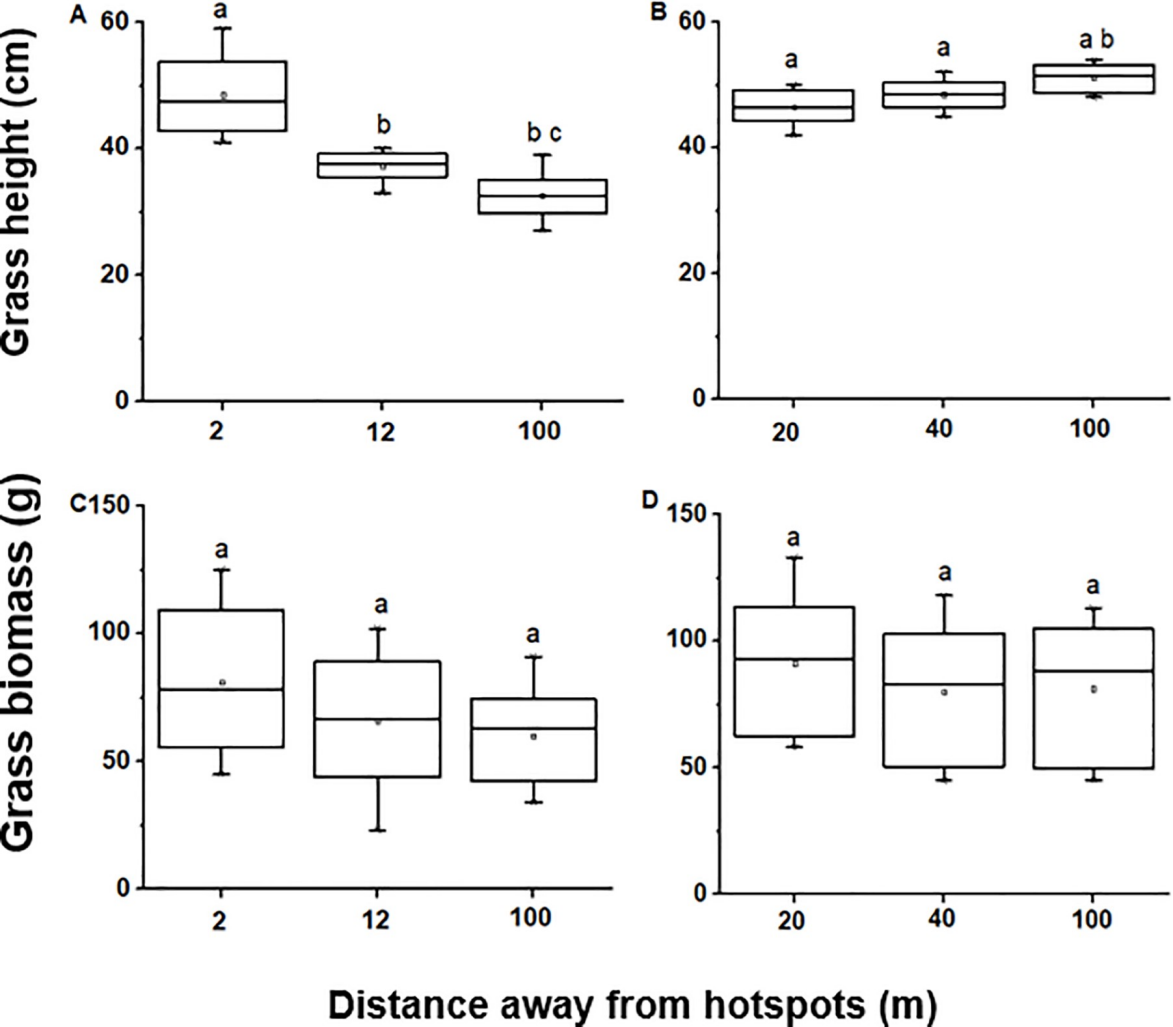
Mean grass height (in cm) and grass biomass (in g) measured as moving away from the influence of termite mounds (A, C) and grazing lawns (B, D), respectively. N = 10 for termite mounds, and N = 6 for grazing lawns. Boxplots show the mean (a square within boxes) and ranges from 25% and 75% quartile (boxes), and the tips of the whiskers indicate standard deviations. Boxes with different letters indicate significantly different means as tested by Fisher LSD at *P* = 0.05.

Grasses from termite mounds were on average almost twice as tall as control grasses and 7% shorter in grazing lawns vs control sites (Tables 1 and 2). With increasing distance away from termite mounds (*F*_*2*, *27*_ = 36.39, *P* < 0.0001), grass height decreased while grass height increased further away from grazing lawns (*F*_*2*, *15*_ = 5.04, *P* = 0.021; Fig 1). Grass biomass did not significantly differ between termite mounds, grazing lawns and their control areas (Tables 1 and 2) nor did it change when moving away from termite mounds and grazing lawns (*F*_*2*, *27*_ = 1.85, *P* = 0.176 and *F*_*2*, *15*_ = 0.301, *P* = 0.744, respectively; Fig 1). Grasses were greener on termite mounds compared to their respective control sites (Table 1) but not in grazing lawns vs control sites (Table 3). GLMM analysis showed that site (hotspot vs control) and location (distance away from hotspot) are the largest contributor to the variation in greenness level (with *F*_*1*_ = 35 and *P* < 0.001 for site and *F*_*1*_ = 119 and *P* < 0.001 for distance). Grass basal cover did not differ significantly between termite mound and control areas nor grazing lawns and controls (Tables 3 and 4). Grass leaf N content of *H*. *hirta* was by 34% higher while soil N content was about two times higher on termite mounds compared to that in control areas (Table 3). Further, GLMM analysis showed a strong interaction between nutrient hotspot location, distance and their interaction with nutrient content (N) (with *F*_*1*_ = 41.95, *P* < 0.001 for hotspot location and *F*_*1*_ = 20.11, *P* < 0.001 for distance). Grass leaf P content of *H*. *hirta* and soil P contents were more than twice as high as those in control areas (Table 3). *H*.*hirta* grass leaf N and P contents in grazing lawns were also about twice as high as those in control areas (Table 4). Furthermore, soil N and P contents were three times and twice as high, respectively, in grazing lawns compared to control sites (Table 4).

### 3.2 Ungulates graze more frequently in hotspots than in the surroundings

**Fig 2:**
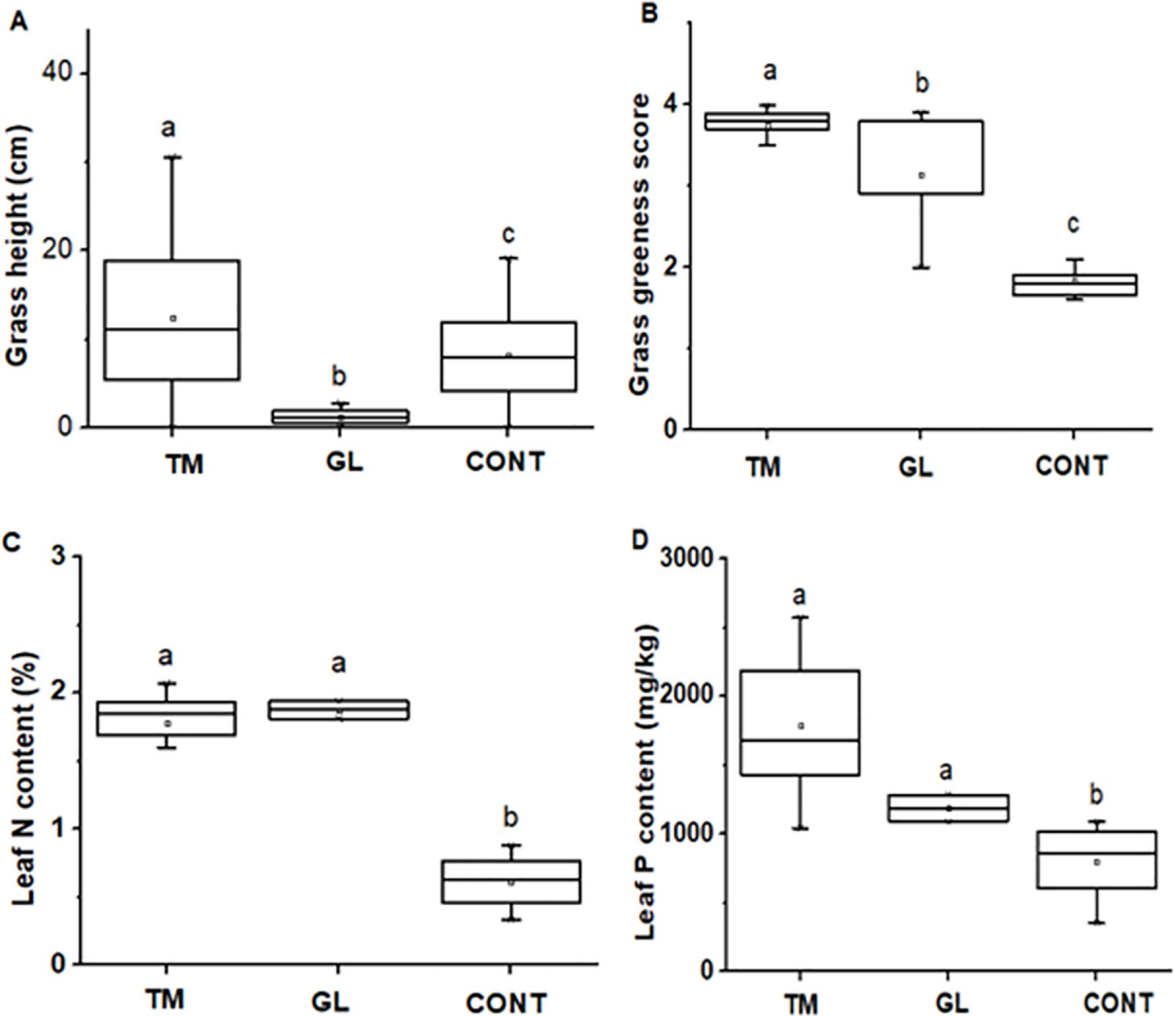
Mean of (A) average *Cynodon dactylon* grass growth height, (B) average greenness score ranging from scorched grass (1), pale green (2), green (3) and deep green (4), Nitrogen (C) and Phosphorus (D) contents in pots containing soils from termite mounds, grazing lawns and control sites after 61 days of the experiment. N = 30. Termite mounds (TM), control (CONT) and grazing lawns (GL). Boxplots show the mean (a square within boxes) and ranges from 25% and 75% quartile, and the tips of the whiskers indicate standard deviation. Boxes with different letter(s) are significantly different by Fisher LSD at *P* = 0.05.

Grass tuft usage estimates decreased significantly with increasing distance from termite mounds (*F*_*2*,*27*_ = 74.17, *P* < 0.0001; Fig 2A). Generally, grass tuft usage signs in termite mound areas were twice as high as those in control areas (*F*_*2*,*18*_ = 123.07, *P* < 0.0001). When moving away from grazing lawns, grass tuft usage decreased (*F*_*2*,*15*_ = 25.68, *P* < 0.0001; Fig 2B), whereby tuft usage estimates around grazing lawns were twice as high compared to controls (*F*_*1*,*10*_ = 192.96, *P* < 0.0001). There was a strong interaction between grass tufts usage and location, distance, season against nutrient contents (N, P) *P* < 0.001. According to our grass preference index analyses, the most frequently consumed species were *H*.*hirta* (scored 88%) and *A*.*gayanus* (scored 64%), while *Sporobolus* spp was not preferred (scored 0%).

**Fig 3:**
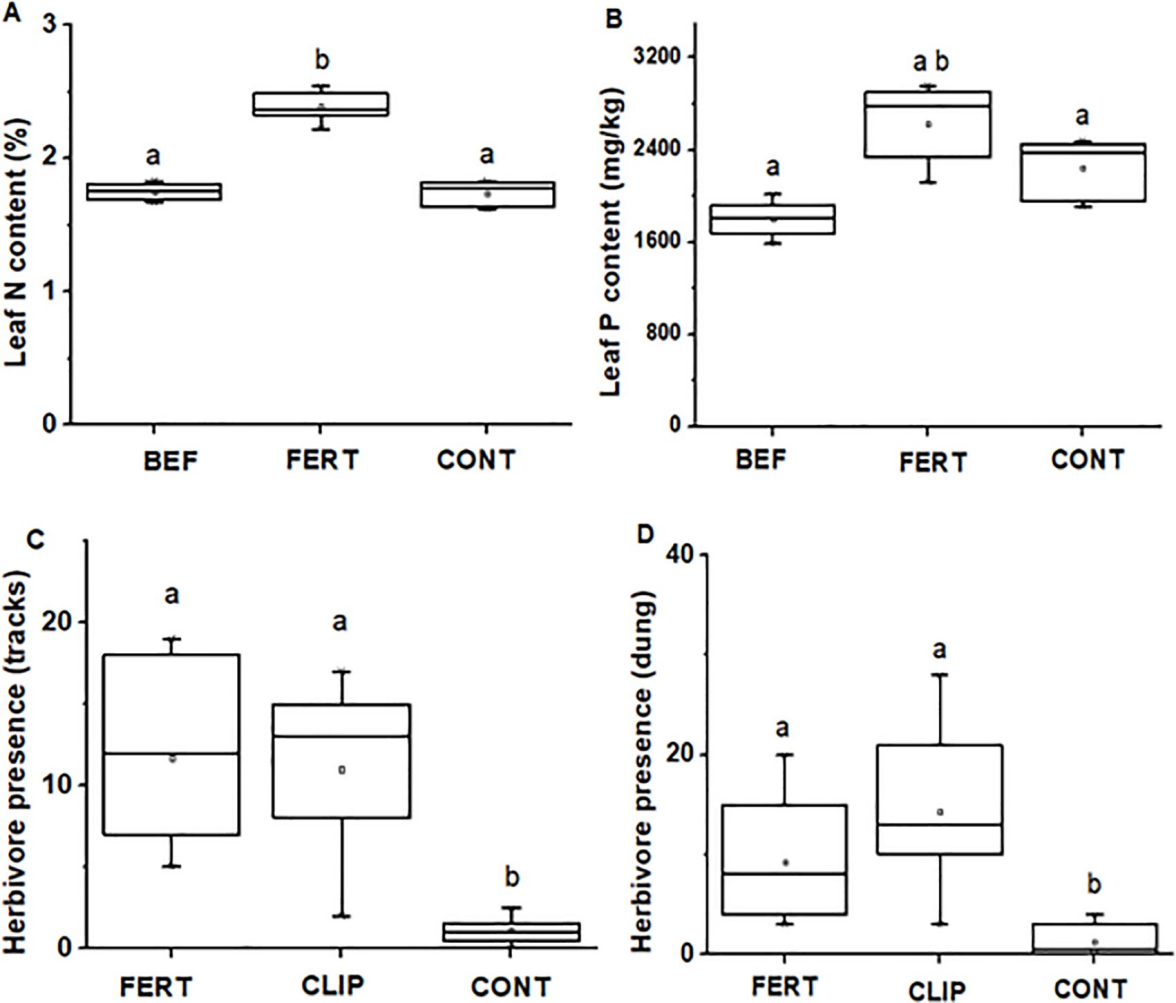
Grass leaf N contents (A) and P contents (B) before setting experimental plots (BEF), in fertilized plots (FERT) vs control (CONT) plots. Herbivore presence using average tracks (C) and dung (D) in fertilized (FERT), clipped (CLIP) and control plots (CONT). Boxplots show the mean (a square within boxes) and ranges from 25% and 75% quartile, and the tips of the whiskers indicate standard deviation. Boxes with different letter(s) are significantly different by Fisher LSD at *P* = 0.05.

Dung and track frequency was seven and 15 times higher, respectively, in termite mound areas than in control areas (*F*_*1*, *22*_ = 10.66, *P* = 0.0035 and *F*_*1*, *22*_ = 8.83, *P* = 0.007; Figs 2C and 5D, respectively). In addition, dung deposition and tracks were three times more frequent in grazing lawns compared to control areas (*F*_*1*, *22*_ = 16.33, *P* < 0.0001 and *F*_*1*, *22*_ = 23.74, *P* < 0.0001; Fig 3A, respectively). The three most frequently observed grazing ungulate species based on tracks and dung were hartebeest, roan antelope and reedbuck (Fig 3B). In termite mound areas, roan antelope was responsible for 78% of the visitation activity (mean tracks and dung deposition), hartebeest for 21%, while reedbuck only contributed 1% (Fig 3B). In grazing lawns, roan antelope was responsible for 30% of the visitation activity, while reedbuck and hartebeest contributed 25% and 44%, respectively (Fig 3B).

### 3.3 Grass appearance and nutrients are the attractant

**Fig 4:**
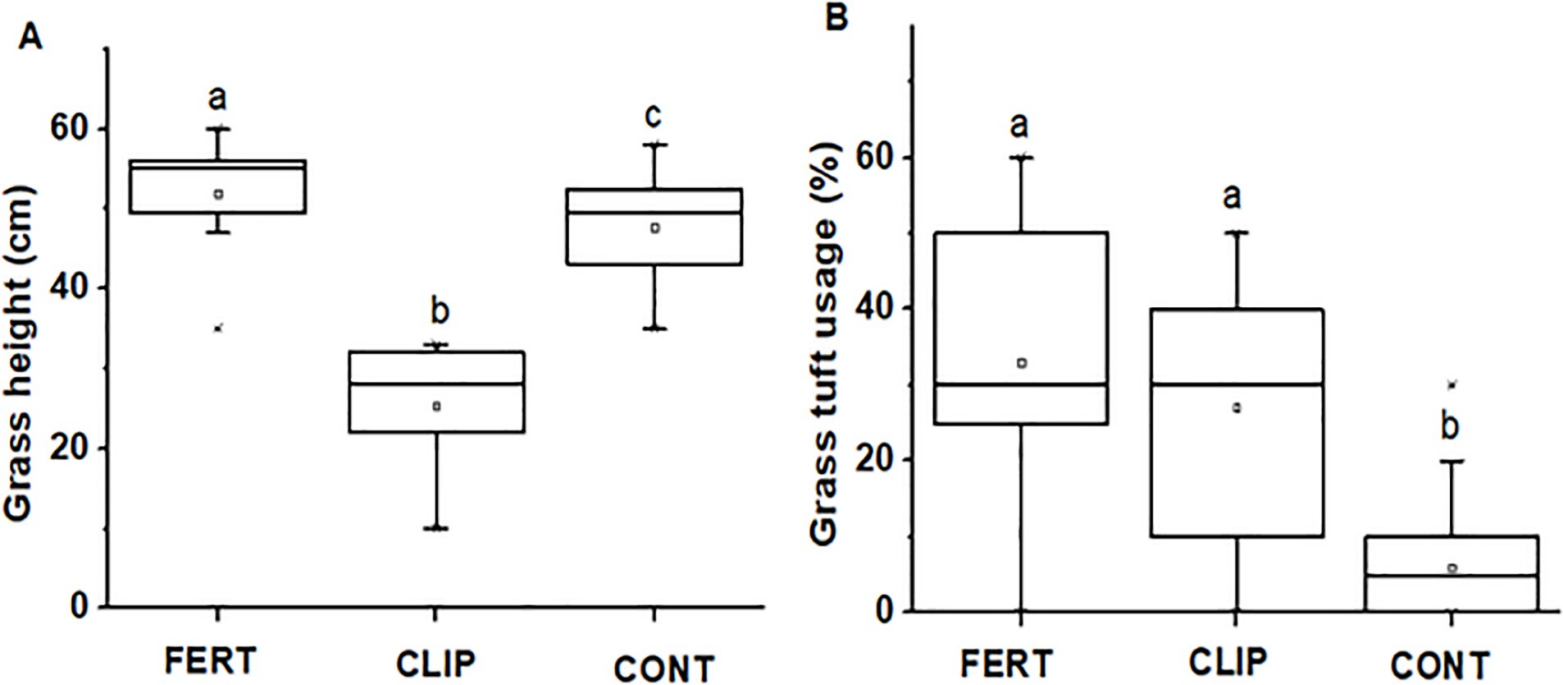
Average (A) grass height and (B) tuft usage between fertilized (FERT), clipped (CLIP) and control plots (CONT). Boxplots show the mean (a square within boxes) and ranges from 25% and 75% quartile, and the tips of the whiskers indicate standard deviation. Boxes with different letter(s) are significantly different by Fisher LSD at *P* = 0.05.

**Fig 5:**
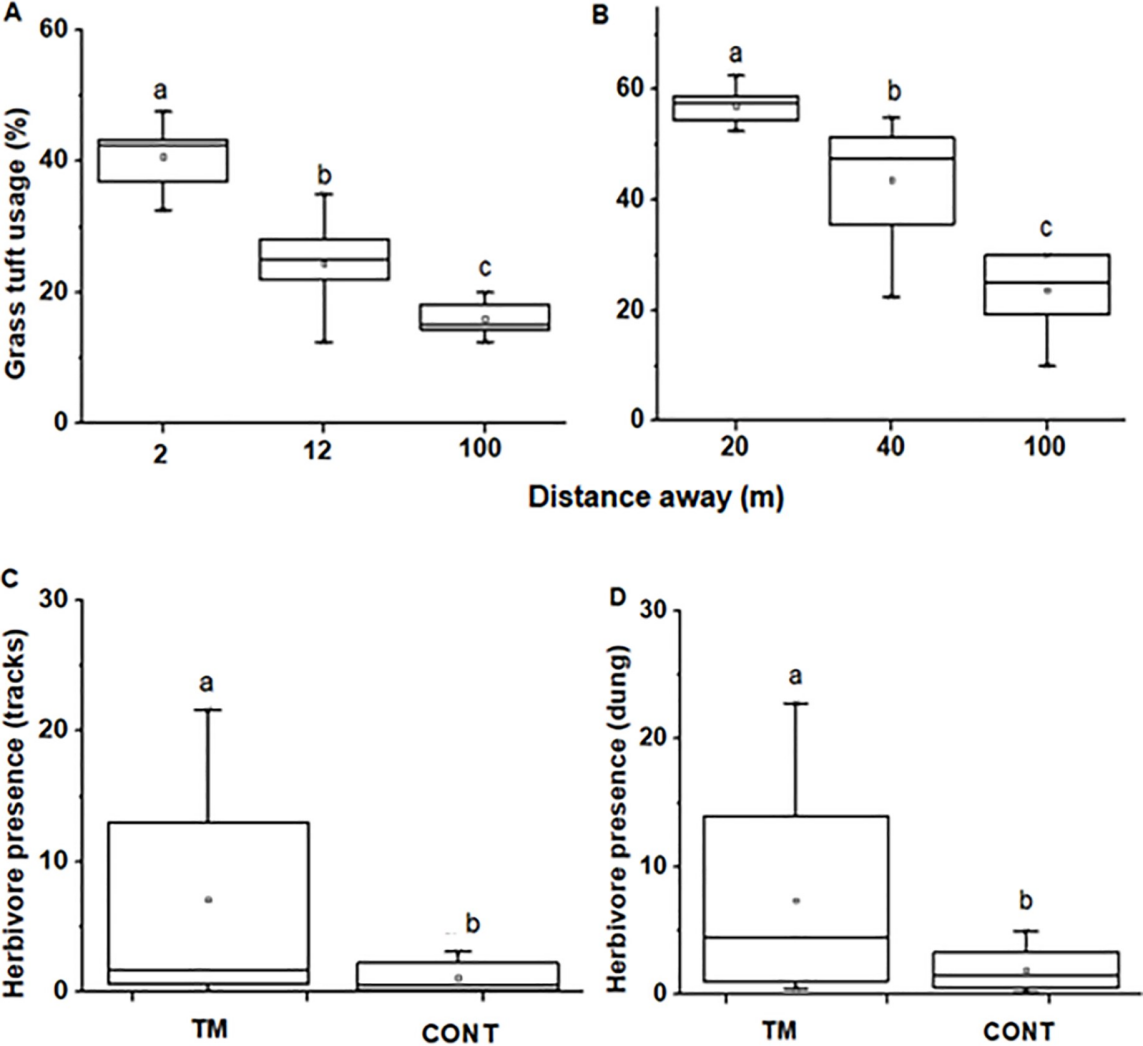
Grass tuft usage estimates in % when moving away from (A) termite mounds (TM) and (B) grazing lawns (GL). Herbivore presence averaged using tracks (C) and dung (D) between termite mounds (TM) vs control (CONT). Boxplots show the mean (a square within boxes) and ranges from 25% and 75% quartile, and the tips of the whiskers indicate standard deviation. Boxes with different letter(s) are significantly different by Fisher LSD at *P* = 0.05.

In the fertilizing and clipping experiment, grass leaf N and P values collected at the beginning of the experiment differed significantly from those collected at the end of the experiment and, as expected, between fertilized and control plots (For N: *F*_*2*, *16*_ = 118.78, *P* < 0.0001, for P: *F*_*2*, *16*_ = 14.91, *P* < 0.0001; Fig 4A and 4B). Our Generalized Linear Mixed Model (GLMM) showed that herbivore tracks and dung were about ten times as frequent in clipped and fertilized plots than in control plots (*F*_*2*, *18*_ = 13.11, *P* < 0.001; Fig 4C and *F*_*2*, *18*_ = 8.44, *P* = 0.0025; Fig 4D). Grasses were on average by about 5 cm higher in fertilized vs control plots (*F*_*2*, *258*_ = 412.46, *P* < 0.0001; Fig 5A). Furthermore, grass tuft was highly eaten in fertilized plots and clipped plots compared to control plots (*F*_*2*, *269*_ = 96.42, *P* < 0.0001; Fig 5B).

### 3.4 Termite mound soils enhance grass growth

**Fig 6:**
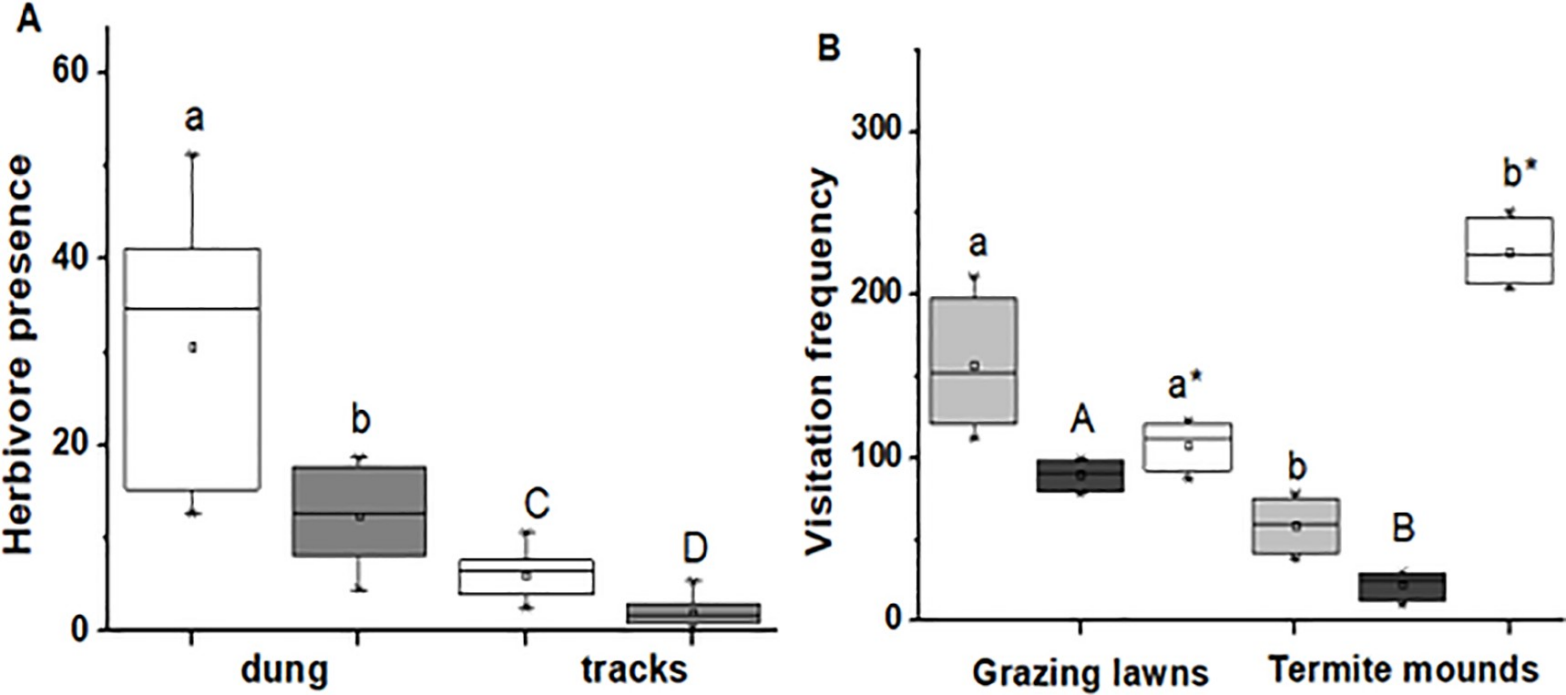
A. Herbivore presence averaged using dung between grazing lawns (white box vs controls (grey box) and herbivore presence using tracks between grazing lawns (white box) vs controls (grey box). Capital letter and small letters has been used to differentiate dung and tracks. B. Herbivore visitation frequency, hartebeest (grey box), reedbuck (black box) and roan antelope (white box) in grazing lawns and termite mounds. Capital letters indicate significant differences between reedbuck visitations, small letters with asterisk compare Roan antelope visitation and small letters compare means of hartebeest visitations. Boxplots show the mean (a square within boxes) and ranges from 25% and 75% quartile, and the tips of the whiskers indicate standard deviation. Boxes with different letter(s) are significantly different by Fisher LSD at *P* = 0.05.

*Cynodon dactylon* height differed significantly across pots with termite mound soil, grazing lawnsoil and control area soil (*F*_*2*,*180*_ = 61.57, *P* < 0.0001, Fig 6A). Termite mound soil grass was 34% greener than control site pots while grasses grown in grazing lawn pots was 24% greener than control sites (*F*_*2*, *180*_ = 296.69, *P* < 0.0001) Fig 6B). Grass leaf N and P contents of *C*.*dactylon* in pots with termite mound soils were three times and twice as high as those from control plots (*F*_*2*, *19*_ = 73.24, *P* < 0.0001 and *F*_*2*, *19*_ = 18.25, *P* < 0.0001, respectively; Fig 6C and 6D).

### 3.5 Seasonal effect impacts grazing activities around nutrient hotspots

**Fig 7:**
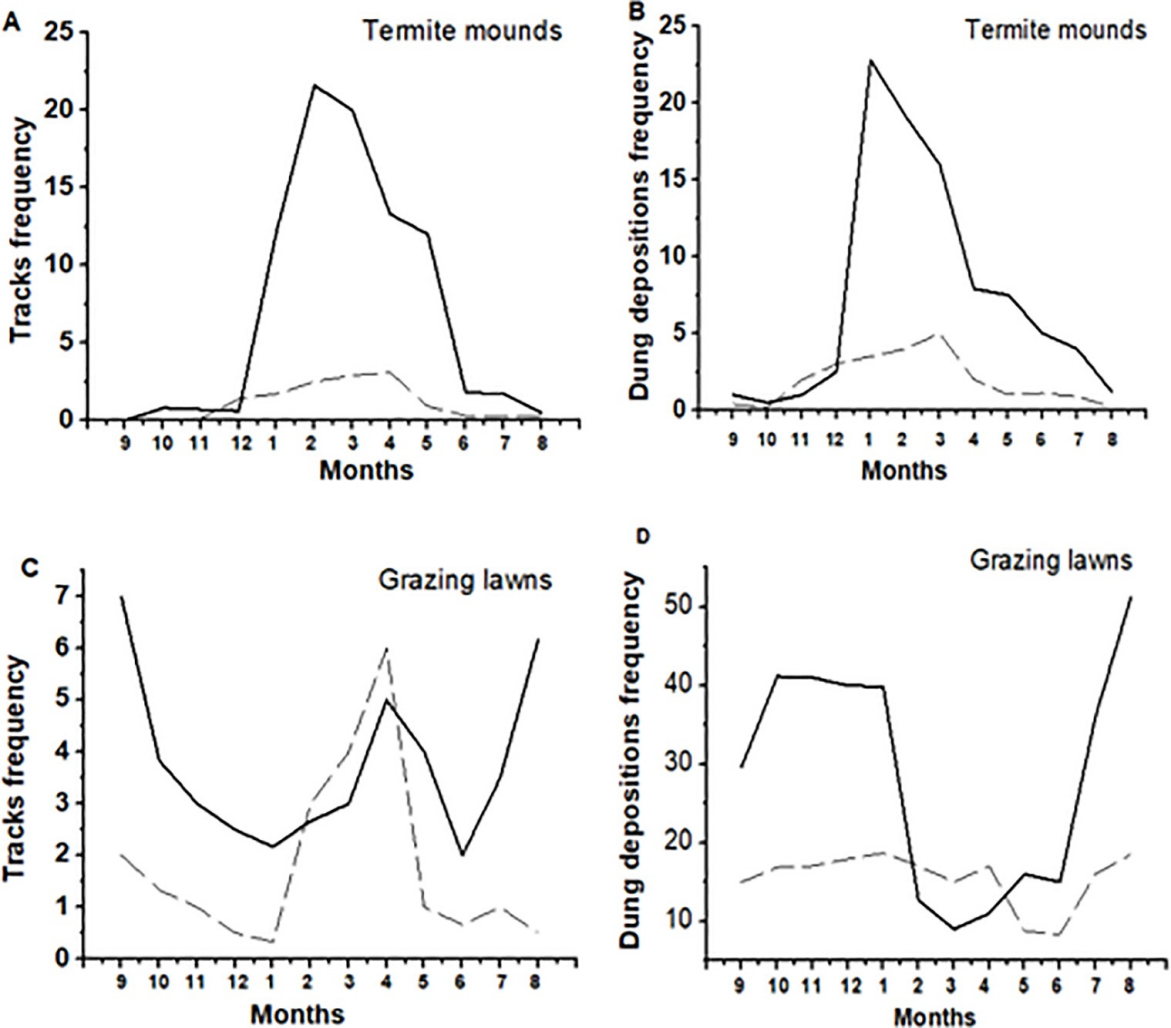
Herbivore presence using tracks (A) and dung (B) on termite mounds (solid lines) vs controls (dashed lines) and tracks (C) and dung (D) between grazing lawns (solid lines) vs controls (dashed lines) over the year.

Dung deposition on termite mounds was four times as frequent during the wet season (*F*_*1*, *10*_ = 10.17, *P* = 0.009; 7 b), while tracks were eight times more frequently observed during the dry season (*F*_*1*, *10*_ = 16.36, *P* = 0.002 (Fig 7A). In grazing lawns, dung depositions were twice as frequent in the dry season than in the wet season (*F*_*1*, *10*_ = 7.13, *P* = 0.02; Fig 7D), while tracks showed no seasonal difference (*F*_*1*, *10*_ = 7.51, *P* = 0.97; Fig 7C). Grass height differed significantly between seasons, with taller grasses on termite mounds during wet season vs shorter grasses during dry season (*F*_*3*, *36*_ = 55.4, *P* < 0.0001), whereas grass tuft usage was higher on termite mounds compared to control areas and grazing lawns compared to control areas (*F*_*5*, *54*_ = 153, *P* < 0.0001) and (*F*_*5*, *30*_ = 64.5, *P* < 0.0001), respectively. Grass biomass was higher on termite mounds and grazing lawns in the wet season compared to the dry season (*F*_*2*, *27*_ = 47.3, *P* < 0.0001) and (*F*_*2*, *15*_ = 5.9, *P* = 0.012), respectively, with season being the largest contributor to the variation in grass biomass (*F* = 245.79, *P* < 0.001).

## 4.0 Discussion

### 4.1 Grass and soil characteristics and hotspots

Our study highlights that nutrient hotspot areas offer important feeding grounds for various grazers in western Tanzania, in accordance with previous studies [1,37]. Grass height around termite mounds was taller than height from control sites, which is similar to studies in South Africa [28,53]. We found no differences in grass biomass between grazing lawns and controls in contrast to [8] and [17] who found that grazing lawn areas showed reduced grass biomass due to high utilization by grazers. In our study, grazer presence was generally low and grazers were rarely seen on grazing lawns, hence they might not have had a great reduction effect on the lawn grass biomass overall [54,55] and grazing lawns might have been created in times when grazers had been more present in that area [56]. Grass species richness around termite mounds differed from control sites, but not for grazing lawns and their control sites, and the most dominant grass species at all sites was *H*.*hirta*. This is similar to [29,57] and [58] who also found differences in species richness between termite mounds vs controls. *Hyparrhenia hirta* was preferred by grazers, which was also reported for grazing ungulates in South Africa, particularly by roan antelope and hartebeest [59,60]. Understanding preferred grass species and locations is important for management as grazers will disperse into preferred hotspots, which can then be predicted [13].

Our onsite experimental plot showed that fertilization enhanced grass greenness and, thus, might have made the grass more attractive for grazers, as our indirect observations suggest. Additionally, termite mound areas had high soil N and P levels, impacting the nutrient levels of grass as well as appearance of the grass. Further, distance away from hotspots strongly impacted grass and soil nutrient contents. Fertilized plots showed the highest frequency of grazer visits compared to their respective control sites, which suggests that elevating nutrient input may attract herbivores [14,61]. Hence, our findings confirm observations on the selectivity of herbivores in other savanna systems, and which clues herbivores use to find appropriate forage [62,63].

### 4.2 Grazing ungulates forage more frequently in hotspots than in the surroundings

We found spatially concentrated dung depositions, which are highly important for nutrient input in soils and elevate grass nutrients, in close proximity to our nutrient hotspots, which will further promote grass quality [16,64,65]. In addition to nutrient input, grazing stimulated fresh regrowth, which occurred on termite mounds during the wet season, further attracting herbivores. This is in contrast to [66], who reported that during the wet season time, grazers spread out for foraging because fresh grass is widely distributed. In the dry season, grazers are under pressure due to nutritionally-deprived forage [8], which is when they visited grazing lawns more often in our study. Despite the lack of green grass in the dry season at Issa, termite mounds were still used as climbing stones for grazers, likely for scanning the landscape for predators (pers.obs). Further, our results showed that grass tufts were found to strongly interact with distance, season and nutrient availability. However, grazing impact is measured subjectively and only into categories but we refer to other studies that have recorded grazing impact like we did [27,32,34].

### 4.3 Grass appearance and nutrients are the attractants

In our study, we found that grazers strongly exploited fertilized plots, a result that is consistent with earlier findings in South Africa [14]. In addition to nutrients, we also found that clipped plots, i.e., with a favorable grass structure and fresh regrowth, were strongly used by herbivores. As grazing lawns are open areas with short and nutrient-rich grasses defined by frequent grazing [4], they easily attract animals, particularly grazers. However, very few experiments have sought to assess the structural and physiological factors that draw animals to resources [14]. Here, we disentangled the factors that might make nutrient hotspots important for mammalian grazers and showed that grass appearance as well as nutrients might contribute to their attractiveness for mammalian grazers. Additionally, spotted hyena *(Crocuta crocuta)* and leopard (*Panthera pardus*) were among the most commonly detected predators in the study area (Piel, pers. obs.) [26], often found on grazing lawns and termite mounds (pers.obs), which might have influenced ungulate distribution and resource use in our study area [67]. In addition, the results of our experiment may have been affected by local people harvesting *Orchid* species in June 2017 close to our sites. Further, a total of 40 plots were affected by wild fires between July and August 2017. This jeopardized longer-term observation on grazing lawn attractiveness. We are aware that our results here provide only a snapshot of ungulate-ecology interactions in the ecosystem. Our grazing lawns were about 1 ha in size (70 x 70 m), while the termite mounds’ influence areas were within a radius of about 30 m [28]. Our results show that grazing lawns were frequently visited, particularly in the dry season, which might be due to their openness [4]. However, termite mounds, despite their small size may also act like small grazing lawns as grazers intensely feeding on their vegetation may increase the nutrient input through their dung over time [14], which in turn might again affect forage selection by grazers [68]. Further, hydrological properties of termite mounds [69,70] in combination with their nutrient properties may further contribute to their important role as hotspots. In the African savanna, termite mounds are conspicuous long lived structures [71], whether active or inactive, and may be occupied by a growing fungus of the species *Macrotermes* [72]. Further, inactive mounds are liable to be recolonized and to become active again at any time [72], hence remain nutrient rich areas.

### 4.4 Termite mound soils enhance grass growth

In our experiment, pots with termite mound soil had the tallest and greenest *C*.*dactylon* individuals compared to other soils, which clearly supports findings of previous work showing that termite mounds are nutrient sinks [1,73] and can strongly enhance grass nutrients and appearance [59]. *Cynodon* spp. is a frequently found grass in Tanzanian savannas and widely used by wild and domestic grazers in eastern Africa [74,75]. Grass greenness, reflecting high nutrient levels [27], might be an important cue for mammalian herbivores [62,76]. Hence, our results show that termite mound soils are highly important attractants for grazers. Pots with grazing lawn derived soils did not store water effectively, which was likely due to the soil type (pers.obs), hence *C*. *dactylon* grass did not grow better, despite the high nutrient content of these soils. Moisture stress and elevated temperature can suppress the growth of *C*. *dactylon* [74], thus rendering our observed grazing lawns unlikely to serve as a nutrient hotspot in the African savanna.

We conclude that in nutrient poor savannas termite mounds and grazing lawns are important in maintaining grass quality for various grazers and that their importance differs across seasons. We further suggest that grazing lawns can be created through clipping and or fertilization, which could be a restoration method especially in fragmented or uniform areas to attract grazers. Central to a better understanding of the role of termite mounds and grazing lawns, there is a need to further understand herbivore population numbers in relation to resource availability in this fragile Miombo ecosystem in the future. We suggest remote sensing for scaling up and mapping resource distribution across various seasons. Additionally, we recommend more studies on fire impacts and possible human disturbances in the study area.

## Supporting information

S1 Fig (DOCX)
