# Supplementary material for: The importance of nutrient hotspots for grazing ungulates in a Miombo ecosystem, Tanzania
Source: PLoS One. 2020 Mar 30;15(3):e0230192. doi: 10.1371/journal.pone.0230192 (PMC7105114; doi:10.1371/journal.pone.0230192)
Supplement: S1 Fig — (DOCX) [file pone.0230192.s001.docx]

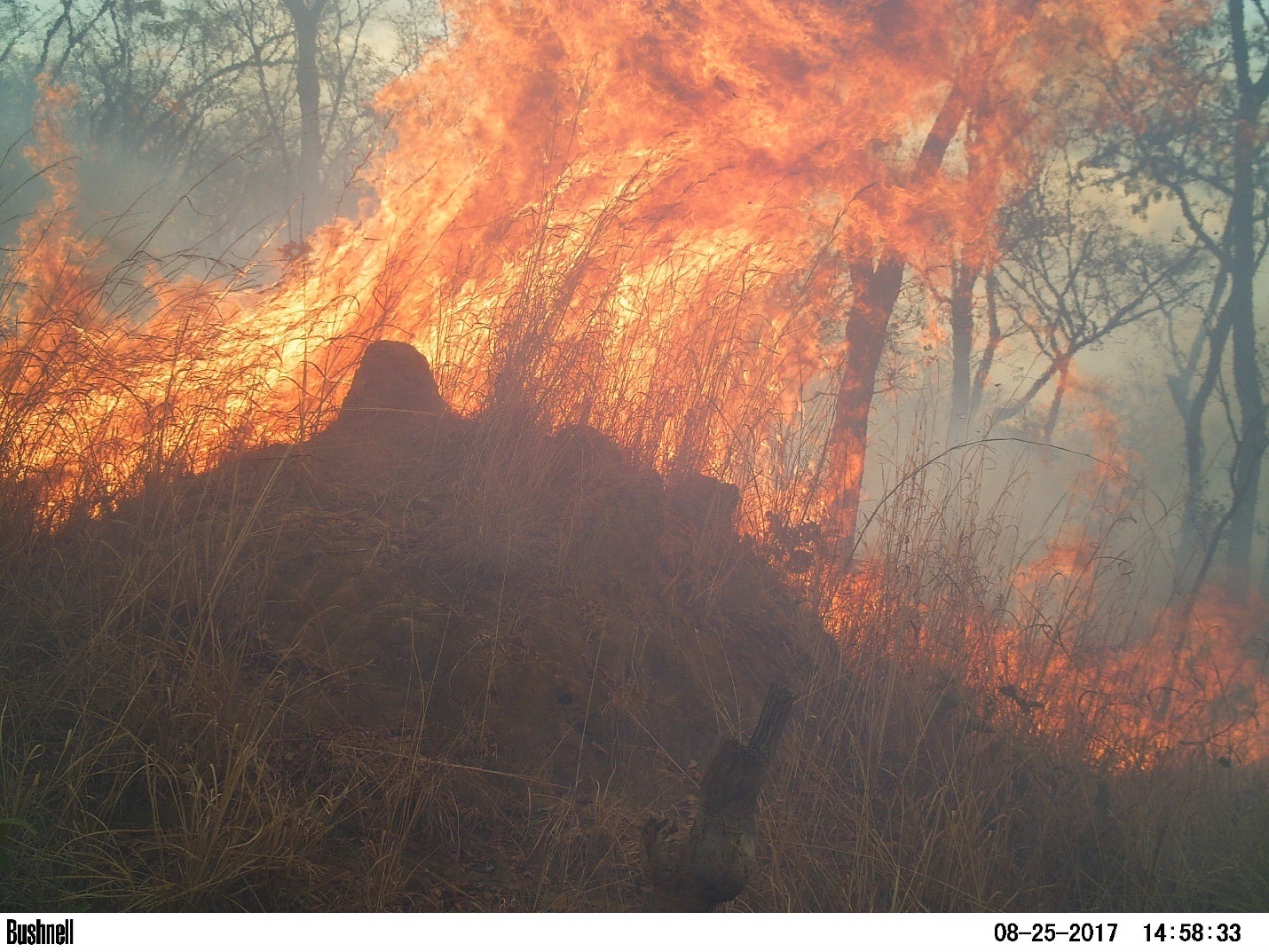


**Image 1:** Termite mound grass burning during the dry season in Issa Valley, Tanzania. These burnt sites could not be sampled for grass analyses in September 2017.


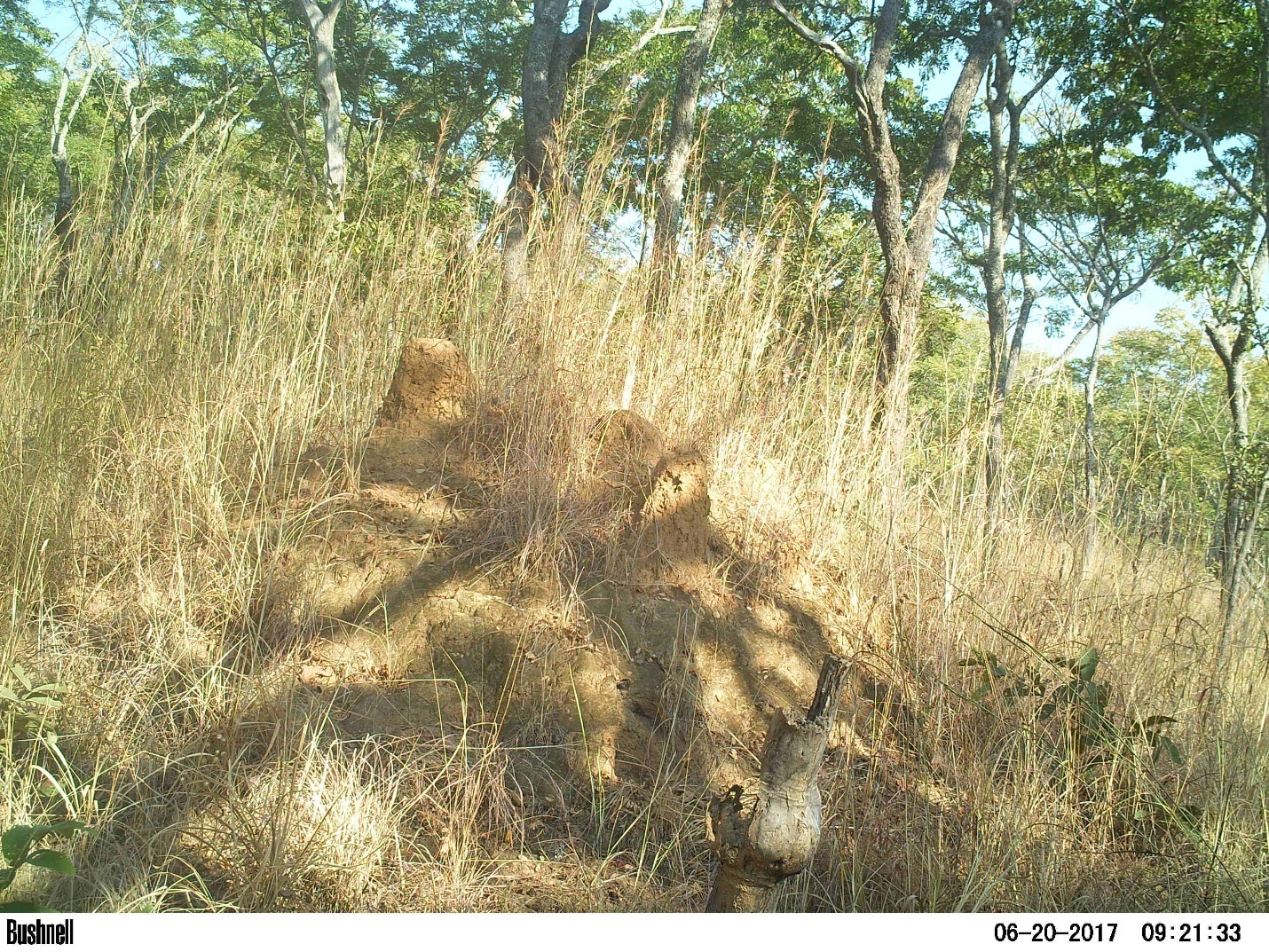


**Image 2:** Termite mound grass during the mid-dry season in Issa Valley, Tanzania.


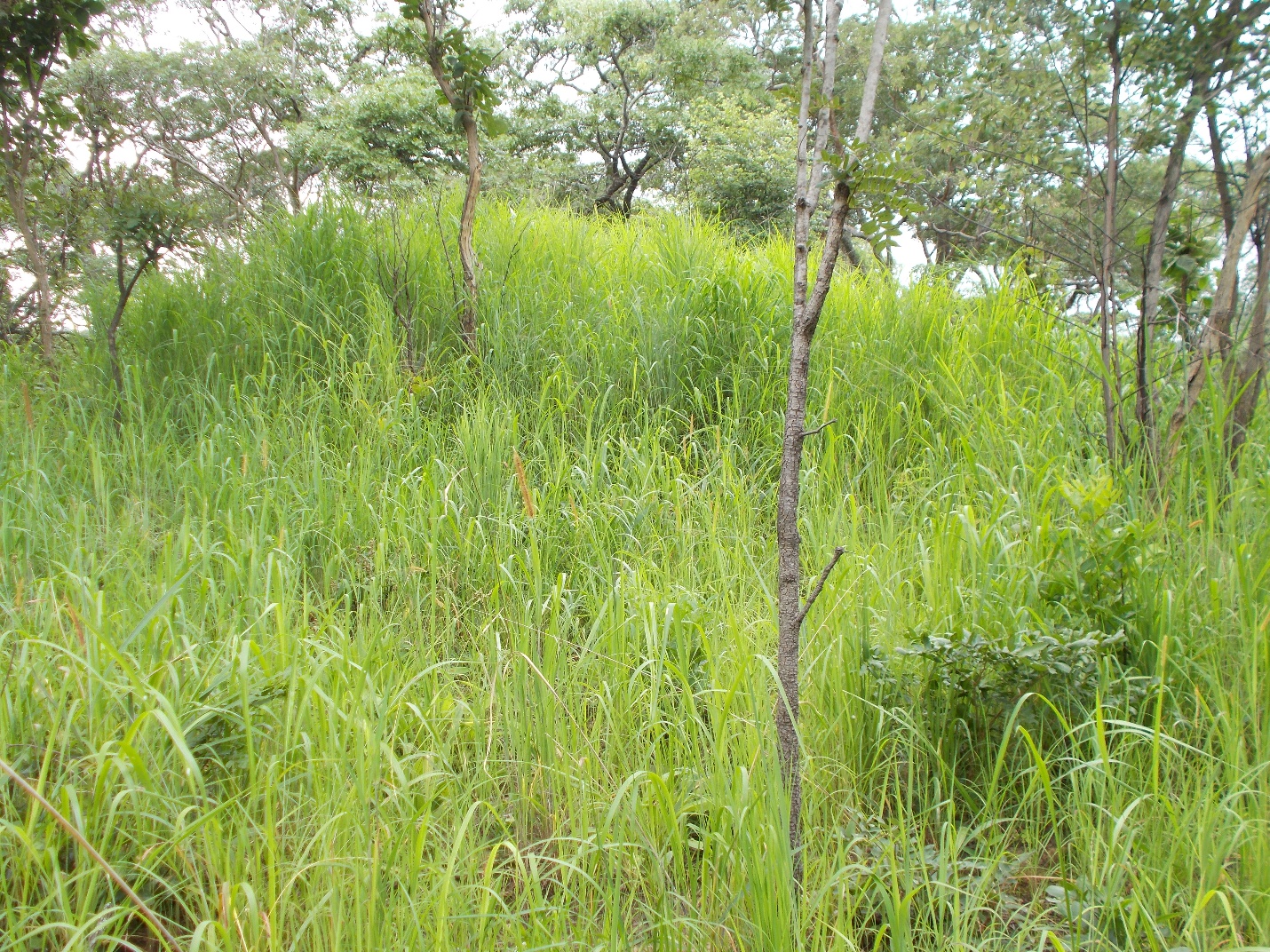


**Image 3:** Termite mound area during the rainy season in Issa Valley, Tanzania.

**
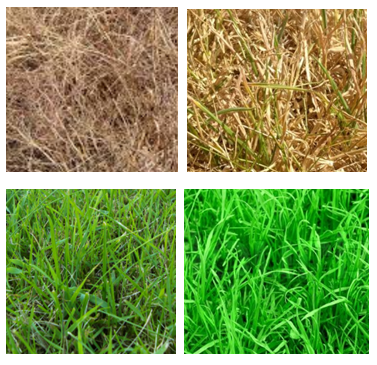
**

**Image 4:** Grass greenness assessment, picture showing different categories of grass greenness which can be identified using different scores: From top left dry grass, top right pale green grass, bottom left green grass and bottom right deep green grass.

Mammal species list to date of animals sighted at Issa valley based on Piel, A. K., Bonnin, N., Amaya, S. R., Wondra, E., & Stewart, F. A. (2018). Chimpanzees and their mammalian sympatriates in the Issa. *African Journal of Ecology*, (October), 1–10. https://doi.org/10.1111/aje.12570

**Artiodactyla**

Lichtenstein’s hartebeest (*Alcelaphus lichtensteinii*)

Roan antelope (*Hippotragus equinus*)

Common waterbuck (*Kobus ellipsiprymnus*)

Klipspringer (*Oreotragus oreotragus*)

Warthog (*Phacochoerus africanus*)

Blue duiker (*Philantomba larvatus*)

Bushpig (*Potamochoerus larvatus*)

Reedbuck (*Redunca redunca*)

Common duiker (*Sylvicapra grimmia*)

African buffalo (*Syncerus caffer*)

Derby's eland (*Tragaphus derbianus*)

Bushbuck (*Tragelphus scriptus*)

**Carnivora**

African clawless otter (*Aonyx capensis*)

Bushy-tailed mongoose (*Bdeogale crassicauda*)

Black-backed jackal (*Canis mesomeles*)

African civet (*Civettictis civetta*)

Spotted hyena (*Crocuta crocuta*)

Serval (*Leptailurus serval*)

African wildcat (*Felis sylvestris*)

Common genet (*Genetta genetta*)

Dwarf mongoose (*Helogale parvula*)

Egyptian mongoose (*Herpestes ichneumon*)

Slender mongoose (*Herpestes sanguineus*)

East African honey badger (*Mellivoria capensis*)

Lion (*Panthera leo*)

Leopard (*Panthera pardus*)

**Hyracoidea**

Tree hyrax (*Dendrohyrax arboreus*)

Yellow spotted/bush hyrax (*Heterohyrax brucei*)

**Pholidota**

Ground pangolin (*Smutsia temminckii*)

**Primates**

Vervet monkey (*Chlorocebus* sp.)

Red-tailed monkey (*Cercopithecus ascanius*)

Blue monkey (*Cercopithecus mitis*)

Senegal galago (*Galago senegalensis*)

Eastern chimpanzee (*Pan troglodytes schweinfurthii*)

Yellow baboon (*Papio cynocephalus*)

Red colobus (*Piliocolobus* sp.)

**Rodentia**

Sun squirrel (*Heliosciurus* sp.)

Porcupine (*Hystrix africaeaustralis*)

Smith's bush squirrel (*Paraxerus cepapi*)

Giant forest squirrel (*Protoxerus stangeri*)

**Tubulidentata**

Aardvark (antbear) (*Orycteropus afer*)
